# On Textual Analysis and Machine Learning for Cyberstalking Detection

**DOI:** 10.1007/s13222-016-0221-x

**Published:** 2016-06-01

**Authors:** Ingo Frommholz, Haider M. al-Khateeb, Martin Potthast, Zinnar Ghasem, Mitul Shukla, Emma Short

**Affiliations:** 1 The National Centre for Cyberstalking Research, Institute for Research in Applicable Computing, University of Bedfordshire, Luton, UK; 2 Web Technology and Information Systems, Bauhaus-Universität Weimar, Weimar, Germany

**Keywords:** Cyber security, Cyberstalking, Cyber harassment, Text analytics, Machine learning, Author identification

## Abstract

Cyber security has become a major concern for users and businesses alike. Cyberstalking and harassment have been identified as a growing anti-social problem. Besides detecting cyberstalking and harassment, there is the need to gather digital evidence, often by the victim. To this end, we provide an overview of and discuss relevant technological means, in particular coming from text analytics as well as machine learning, that are capable to address the above challenges. We present a framework for the detection of text-based cyberstalking and the role and challenges of some core techniques such as author identification, text classification and personalisation. We then discuss PAN, a network and evaluation initiative that focusses on digital text forensics, in particular author identification.

## Introduction

Personal threats, false accusations, privacy-violation and defamation are typical forms of attacks faced by victims of harassment and stalking. The advancement in Information and Communication Technology (ICT) has extended existing attack vectors further to include many online social-networks designed for people to interact using multimedia. Content forms such as text, images, audio and video are utilised in the context of Human Computer Interaction (HCI) methods to interface with end users. This is usually enabled through web browsers, mobile applications and other such means.

The significant impact of ICT on the severity of cyberstalking has been reported in the literature. For instance, research from the Electronic Communication Harassment Observation (ECHO) project [23, 31] shows that many incidents, although initially emerging in cyberspace, have consequently moved to the physical world. Extreme examples of such incidents have forced victims to disengage from their daily routines, move homes, and/or change jobs resulting in significant financial losses, inducing fear, distress, and disrupting the daily activities of victims. Accordingly, terms such as cyberstalking and cyberbullying have emerged to address the problem with full consideration of the heavily interconnected Cyber-Physical-Natural (CPN) world [18] to accurately define the ecosystem where both victims and attackers practice all their life-related activities. There is evidence that the extreme emotional distress and physical trauma caused by these anti-social offences have also led to suicide and murder [31].

It is important to elaborate on the unique characteristics of cyberstalking; in this paper we define cyberstalking messages to be: 1) unwanted or unwelcome; 2) sent from a known or unknown but determined/motivated party (perpetrator); 3) intentionally communicated to target a specific individual (the victim), and 4) persistent. The National Centre for Cyberstalking Research (NCCR)^1^, based in the UK, further recognises the persistent behaviour to be realised when ten or more unwanted messages are sent over a period of time that is equal to or less than four weeks. Clearly, this discussion sets a distinctive line between cyberstalking and any discrete events of online harassing materials. To effectively mitigate the risks associated with cyberstalking, technology must be utilised to support detection, event classification, automated responses, and reporting of incidents. Text analysis and Information Retrieval (IR) play a critical role given that text (emails, SMS, instant messaging (IM), Blog posts, Twitter tweets, etc.) is a popular content form reportedly used in the vast majority of incidents.

In the remainder of this paper we elaborate further on the need for technical solutions to tackle cyberstalking. Afterwards, in Sect. 3, we discuss a framework for cyberstalking detection and evidence gathering; this also includes the application of text analysis and machine learning in this context. One of the main emerging challenges within technical solutions is authorship identification. We therefore also introduce a the PAN shared task series that tackles this task in Sect. 4. It is one of the aims of this paper to relate this line of research to the context of automatic solutions to detect and handle cyberstalking in text messages.

## Finding solutions to curtail cyber harassment and cyberstalking

The importance of an adequate cyber crime response is recognised to be a cross-cutting issue in cybersecurity and law enforcement as it has clear links to serious organised crime, protecting the vulnerable and victims of child sexual exploitation [37]. The growth of the internet has led to the traditional crimes of stalking and harassment being transformed in scale and form. Much of the recent research into cyberstalking has focussed on the comparisons between offline stalking and cyberstalking and the mental health outcomes of the victims of stalkers. Therefore, the necessity to increase understanding of the technological means of detecting and gathering evidence in cases of cyberstalking is paramount.

Although there is no conclusive evidence as to the increasing prevalence of cyberstalking on account of advancements in technology, it can be assumed that the number of cyberstalking incidents has indeed risen dramatically. According to a report released by the UN’s International Telecommunication Union (ITU) in 2013, approximately 39 % of the world’s population now has access to the internet, which is equivalent to around 2.7 billion people. Online resources can also be utilised unlawfully by criminals. For instance, with regard to cyberstalking, criminals have an infinite number of online users to stalk or harass.

From a broad perspective, the issues surrounding and the consequences of acts such as cyberbullying, harassment, and stalking are most certainly within the public’s *zeitgeist*. For example, TV shows and films are increasingly produced on this subject matter and not necessarily from a fictional standpoint. However, realistic solutions are rarely forthcoming beyond the required narrative closure. Legally, since cyberstalking is a criminal offence in some countries, the system partially contributes to the solution. For instance, in the UK, based on the circumstances of a given case, relevant laws could include the Sexual Offences Act 2003 S.15, Protection from Harassment Act 1997, Crime and Disorder Act 1998, and Domestic Violence, Crime and Victims Act 2004. Additionally, a number of support services (e.g. The National Stalking Helpline) provide the community with advice on how to report harassment, gather evidence and reduce risk. However, relevant technologies have only been very briefly researched to produce applicable solutions. Therefore, beyond advice on best practice (for example see [25]) for those who find themselves as the target of cyberstalking and the like novel technical solutions are needed. These technical solutions are needed not only from a prevention or evidenciary basis but also so that those who find themselves as the focus of these types of attack can feel a sense of regaining control, loss of control being one of the many consequences as reported by the ECHO project [23, 31].

Current literature includes proposals aiming to shield unwanted communication; provide training and emotional support through simulators; and facilitate incident reporting and digital investigations [20]. As communication channels are hard to control, current proposals in this area suggest a layer of encryption and integrity checking to preserve privacy and facilitate identity checking [10]. This will presumably prevent unwanted communication but the scenario adopts a white-list approach where each connection is pre-approved. This can be very efficient within a parental-controlled environment to protect minors but not convenient for adults with extensive online tasks to perform as part of their career or social life. A good solution should ideally empower users with real control over unwanted messages without restricting their online reachability. Other existing methods utilise traditional techniques to restrict contact (e.g. block IDs and mobile numbers); although attackers in many stalking scenarios are known to the victims, this approach still fails due to the high-degree of online anonymity possible in cyberspace, for instance, perpetrators can forge email headers, create new social media accounts, and hide their IP addresses via Privacy Enhancing Technologies (PET) [16].

Reactive proposals are focused on incident response, usually through digital investigation toolkits designed to not only recover the attacker’s identity but to preserve an admissible evidence to a court of law. Software such as the Predator and Prey Alert (PAPA) system [2] enables remote monitoring of local activities by the police to facilitate investigations and collect evidence; such solutions require an agent software to be installed at the end-user’s side to be able to also monitor encrypted communications [4]. Clearly, this comes at a price in terms of user privacy but could be effective in many extreme cases. In response to online anonymity, authorship analysis can be performed to establish hypotheses on which content belongs to which user. Eventually, determining particular details such as the age, gender or physical location from contextual clues can help a system to automate a response (e.g., warning, block, report) [3]. Nonetheless, content forms can sometimes be directly linked to the originator, for instance pictures can be associated with the particular camera it was taken by, or to other images produced by the same camera. This has been tested based on the analysis of Sensor Pattern Noise (SPN), published results suggest a satisfactory outcome for this technique [29].

An example of the other type of reactive responses could be initiated through peer-support simulators, a virtualised application to provide social services including emotional comfort and standardised professional advice to victims [39]. Likewise, proactive solutions could include training through simulators or by means of serious games [8] to educate and raise awareness of the problem.

An important conclusion from these examples is that automated detection through machine learning and text analysis is a fundamental component to provide intelligence in each case. Detection is known to be the first step to trigger a suitable action and mitigate the incident; alert a supervisor, block communication, and preserve evidence. As such, this is currently an active research area at a very early stage where data mining algorithms are trained, pre-processing techniques tested, and new corpora are being built [14, 7, 9].

The prior techniques were discussed to demonstrate a practical response. While their combination yields a promising plan customised to mitigate cyberstalking, the applicability of such implementations can also be extended to cover other forms of cybercrime. For instance, the functionality of a mobile application designed to report harassment could, in theory, be generalised to consider blackmail, fraud and other anti-social behaviour. The Crown Prosecution Service in the UK categorise criminal offences sent via social media into credible threats that could constitute violence to the person or damage to property; targeted attacks on individuals such as Revenge Porn; communications which may amount to a breach of a court order or a statutory prohibition, and finally, communications which are Grossly Offensive, Indecent, Obscene or False [36]. Any digital evidence created using media recorders could be shared with law enforcement using the same process but using different methods. Likewise, any empirical results on the feasibility of using simulated agents in virtual reality to mitigate cyberstalking by means of training and social support, would trigger advancement in the field to provide algorithms modelled to automated conversations with people suffering depression, anxiety disorder, or even eating disorders.

## A framework for automatic cyberstalking detection in texts

Having discussed the need for technical solutions to tackle cyberstalking, we now turn to the question how different text analysis, information retrieval, and machine learning techniques could be utilised to detect potentially harmful cyberstalking messages and to collect the required evidence for law enforcement. To this end, we present in this section a framework that outlines potential tasks and solutions as well as their relationships. In this respect, author identification is one of the core tasks that undergoes increasing popularity in the research community. We therefore continue our discussion in Sect. 4 where the PAN network on digital text forensics is introduced, which combines different research efforts in this field.

**Fig. 1:**
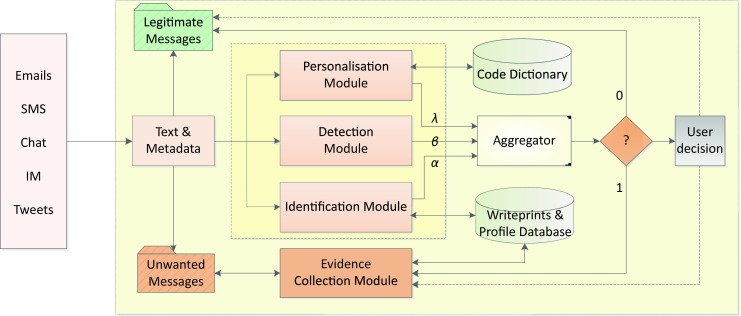
The ACTS framework. Different text analysis and machine learning modules, based on user profiles, content and writeprint/author identification, are used to determine whether a text message is legitimate or unwanted

Our proposed framework is called Anti Cyberstalking Text-based System (ACTS). The framework is a generalisation of the one proposed in [12] (from email to general text messages), which is missing a personalisation module motivated below. It is furthermore adapted from work presented in [11]. The framework proposed here could best be described as a detection and digital readiness system, which specialises in an automatic detection and evidence collection of text-based cyberstalking (e.g., in emails, MMS, SMS, chat messages, tweets, social media updates, instant messages). A prototypical implementation of the framework is under development, and the data collection process is ongoing. ACTS is designed with the aim to run on a user’s computer/mobile device to detect and filter text-based cyberstalking. The architecture of ACTS is depicted in Fig. 1.

The proposed system combines several techniques to mitigate cyberstalking. It consists of five main modules: attacker identification, detection, personalisation, aggregator, and evidence collector.

When a new message arrives, metadata-based blacklists may be applied to filter messages coming from unwanted senders. Such metadata may for instance consist of the header information in emails or the sender in tweets. However, some systems allow for forging such metadata, for instance by providing a fake ‘sender’ header in emails or using anonymous or fake accounts. For this reason, messages that pass the blacklist need to be further examined by the identification, personalisation, and detection modules. The results from three modules are passed to the aggregator for a final decision.

Similar to other email filtering systems (like spam detectors), the detection module is employed to detect and classify messages into cyberstalking messages, genuine messages, and grey messages based on their (textual) content. A number of supervised and unsupervised machine learning algorithms can be employed to classify and filter unwanted text messages [30]. To this end, we assume the detection module computes a value $\beta$ that covers the content-based estimate of the system that the message is unwanted (for instance, based on unwanted words or phrases). A challenging task is to take into account the nature of messages from short SMS and chat messages to potentially longer emails.

Unlike the detection module, the attacker identification module analyses messages based on sender writeprints (in an analogy to fingerprints); these are writing-style features such as structural, lexical, syntactic, and content-specific features [38]. Applying means for authorship attribution and verification (further discussed in Sect. 4), this module is deployed specifically to detect and uncover anonymous and spoofed messages which are sent by a known attacker who may not be detected based on metadata. Furthermore, the evidence provided by the attacker identification module helps classifying those messages which potentially could bypass the detection module as they do not contain any unwanted words or phrases. However, authorship attribution on short messages poses specific challenges for instance due to the character limitation of short messages (e.g., SMS is limited to 160 characters per message and similar limitations hold for tweets). Nevertheless, because of their character limitation, people tend to use unstandardised and informal language abbreviations and other symbols, which mostly depend on user’s choice, subject of discussion and communities [13], where some of these abbreviations and symbols could provide valuable information to identify the sender. A possible solution to overcome this shortcoming and to enhance the identification process is a combination of cyberstalker’s writeprints with their profile, including linguistic and behavioural profiles, utilising already collected writeprints and profiles stored.

The result of the identification module is represented by the value $\alpha$, for instance, based on three outputs: not cyberstalking ($\alpha\geq r_{2}$), cyberstalking ($\alpha\leq r_{1}$) and grey ($r_{1}<\alpha<r_{2}$). The $\alpha$ value is passed to the aggregator component, $r_{1}$ and $r_{2}$ are pre-defined threshold values in attacker identification (which have to be determined empirically).

Cyberstalking and cyber-harassment are abusive and threatening attacks; however, the concept of what is considered abusive and threatening in a message is a subjective decision from a victim’s perspective; we have to take into account that such a decision is a highly personalised one. For example, bare words or phrases in a message might have no inclination whatsoever towards bad feeling to almost anyone, but they might cause fear and distress to a cyberstalking victim. For instance, sending child birthday wishes may commonly be considered as positive, but not in case of somebody who lost their child or had undergone abortion. This complicates the process of developing a general tool to combat text-based cyberstalking. For this reason we define a personalisation module which is employed to enhance the overall victim’s control over incoming messages, where each victim can outline and define their own rule preferences. Therefore the personalisation module may consist of rule based components and a code dictionary. The rule based component is optional, where rules are defined based on words, dates and phrases provided by the user. For example, a typical rule might be $\mathit{if}((\mathit{date_{A}}<\mathit{currentdate}<\mathit{date_{B}})\wedge(\textit{message\ contains\ ``happy\ birthday''}))\textit{\ return\ true}$. If cyberstalking involves ex-partners, the cyberstalker has background knowledge about victims and knows which words/phrases at which specific times can cause distress and fear to victims (in this case “happy birthday”). Furthermore, consider the above example where somebody gets birthday wishes for a lost child; they will likely occur around the time of the actual birthday, hence specifying a time range would make sense as a further means to personalised cyberstalking detection.

A code dictionary is created from ranked words and phrases which are commonly used in cyberstalking. Furthermore, the code dictionary could also be updated by the user. The ranking value for each word and phrase is initially set to zero. Then each time a word or phrase in a dictionary is matched with words or phrases in received message, the ranking value of the matched word/phrase in the code dictionary is increased. Obviously, the most common words or phrases will be ranked highest, and the messages first matched with highest ranked words and phrases.

The received message could be preprocessed; for this purpose k-shingling [6] could be utilised. Shingling is another way to represent features (terms) of a message, which has been used in email classification. A shingle of a message is a sequence of all words in that message; the size $k$ of a shingle is the number of words in that shingle (denoted by $k$-shingle). If a message $m$ can be presented by a sequence of words ${w_{1},w_{2},\ldots,w_{n}}$ then $k$-shingling of $m$ will result in $j$ features with $j=(n-k)+1$, so each feature will cover $k$ terms. For example [6], if we select 4‑shingling ($k=4$) and the message is “a rose is a rose is a rose”, the features are based on (“a rose is a”), (“rose is a rose”), (“is a rose is”), (“a rose is a”), (“rose is a rose”). Where each $k$-length shingle is run against the dictionary, probabilistic disambiguation [1] is another possible method to be used. This is a probabilistic technique used to measure usage violence and extremist hate effects on different online messages. Therefore, such technique could be used to measure the degree of offensiveness and seriousness of cyberstalking messages in relation to a dictionary code database.

Both the dictionary’s returned result and rule-based result are represented by the value $\lambda$, which may be for instance either cyberstalking (1) or not cyberstalking (0) (when both returned results are negative). The final decision whether a received message is cyberstalking or not is made in the aggregator module, utilising the outcome from the previous modules. $\alpha$, $\beta$ and $\lambda$ are the final calculated result values for each individual received message by the identification, personalisation, and detection module, respectively. Messages are identified as either grey (?), cyberstalking (1) or not cyberstalking (0) based on these values. If messages are classified as grey, the respective message may be flagged and the final decision should be made by the user.

The final module is the evidence collection module, which collects evidence from a newly arriving cyberstalking message, for instance, apart from the provided metadata and content, in the case of email the source IP address or, if it is not available, the next server relay in the path, and the domain name (both addresses are automatically submitted to WHOIS and other IP geolocation websites). The information with timestamp and email headers is saved, for instance, in the evidence database on a victims’ device. The module should also regularly update and add stylometric profiles and related information of the cyberstalking message to the database. Furthermore it should utilise statistical methods like multivariate Gaussian distribution and PCA to analyse the writeprint and profiles of cyberstalking, and text mining to extract similar features, attacker behaviour, greeting, farewell, etc., specifically between anonymous messages and non anonymous ones.

Saving cyberstalking messages and evidence locally or in a (private or shared) cloud is another function of the evidence module. This process will allow law enforcement to have regular access to messages as well as have an overview of the cyberstalking progress. Saved cyberstalking messages could be a first step in collecting data on cyberstalking. However, saving data (evidence and emails) is usually an optional function of the system, that would only take place when the victim agrees with law enforcement to save data so that law enforcement could have a regular access and monitor cyberstalking incidents. The process of saving cyberstalking messages, for instance, in a cloud requires some safeguarding to preserve the messages’ integrity and authenticity and protect it from any malicious act (which might destroy or manipulate potential evidence). Hash functions like SHA could be utilised to make sure the exchanged data is not modified during transmission or by any unauthorised person. Furthermore, asymmetric keys could be used for data encryption. Provided a suitable API is available as well as corresponding legislation is in place (e.g. Germany’s ‘quick freeze’ data retention approach^2^), the evidence collection module could also notify the service or content provider.

## Digital text forensics for identification

**Fig. 2:**
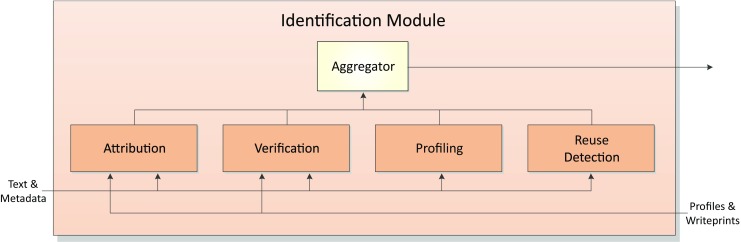
Identification module of the ACTS framework. The module comprises components for various relevant digital text forensics tasks that are used to collect evidence against suspects

An important part of our framework to detect cyberstalking is the author identification module. Its purpose is the analysis of arriving messages with respect to authorship and originality. Figure 2 gives an overview of its four major components, namely attribution, verification, profiling, and reuse detection. Each of these components is invoked under specific circumstances, sometimes in parallel, to collect evidence about the origin of a given message or a given collection of messages. Its results are aggregated and then returned to the surrounding framework. In what follows, we briefly explain these components and their underlying problem settings, we outline their relevance to detecting cyberstalkers, and we point to state-of-the-art research for each of them, much of which originates from a number of shared task competitions that have been organized as part of PAN workshop series on digital text forensics.^3^

For the detection and subsequent prosecution of cyberstalking, it is important to collect evidence on the suspect perpetrators. The application of forensic software for author identification may aid in this respect by comparing the messages received from a stalker with other pieces of writing from a suspect, or that of a number of potential suspects. In cases where the stalker attempts to stay anonymous, this may help in revealing their identity. However, even if the stalker apparently acts openly, collecting evidence that connects the stalker’s messages to the apparent identity of the stalker is an important part of an investigation, since the stalker may try to deceive the investigators. In this connection, technologies for authorship attribution and verification are required to scale future investigations, which, when given a text of unknown authorship, either attribute it to the most likely author among a set of candidates, or verify whether the text has been written by the same author as another given text. The former task corresponds to a traditional task in forensic linguistics, where investigators first narrow down the set of candidates who may have written a given piece of text using other evidence, and then employ a forensic linguist to determine who of the candidates probably wrote the text in question based on stylistic analyses. This presumes of course that suspect candidates can be identified and that sufficient writing samples from each of them can be gathered. In that case, attribution boils down to a multi-class classification problem, where each suspect candidate corresponds to a class. By contrast, verification corresponds to a so-called one-class classification problem [21, 35]: either a text has been written by a given author (the target class), or not, whereas determining the latter would mean to be able to accurately distinguish the given author from all others. While being more challenging to solve automatically, verification problems may frequently arise within cyberstalking detection. For example, one may wish to check whether a message received from a given sender was indeed written by that sender by verifying whether that message corresponds stylistically to messages previously received from the same sender. Altogether, attribution and verification address complementary problem settings within cyberstalking detection.

In situations where little is known about the originator of an offending message, however, neither attribution nor verification technologies are of much use. Here, author profiling technology can be applied to determine at least some demographics about the author of the message in question. Author profiling technology attempts to correlate writing style with demographics, such as age, gender, region of origin, mother tongue, personality, etc., which is typically cast as a multi-class classification problem. This information may help to narrow down the search for suspects. At the same time, author profiling technology may also be used to verify whether the supposed age of the sender of a stalking message is consistent with the results of an automatic analysis, which may raise a flag, or serve as sufficient reasons to doubt the obvious in an investigation. An analysis of personality types may further allow for recommending ways to deal with a supposed stalker in order not to encourage them further.

The automatic assessment of messages with respect to authorship presumes that they have actually been written by their senders. This assumption may not hold under all circumstances; especially when offenders become aware of the fact that their messages are being analyzed with regard to writing style, they may attempt to obfuscate them. While it is still unclear how well humans are capable of adjusting their own writing style so that forensic software or even a human forensic linguist are misled, an easy way to send messages devoid of one’s own writing style is to reuse someone else’s writing. This is why reuse detection forms a integral part of forensic analysis, where the task is to identify texts or text passages that have been reused, and to retrieve their likely sources. Nevertheless, even in the absence reference collections to compare a given message with, a writing style analysis of a message may still be useful, namely to identify writing style breaches (i.e., positions in a message where the writing style changes), which would serve as evidence that texts from different authors have been conflated [34].

All of the aforementioned authorship-related tasks, with the exception of reuse detection, are basically addressed using machine learning applied on top of stylometry, the science of quantifying the writing style of texts. The first application of stylometry to tackle an authorship dispute dates back to the 19th century [24], and since then linguists have proposed plenty of features for this task [17]. In general, such features attempt to capture writing style at character level, at the lexical level, at the syntactic level, at the semantic level, and dependent on the application. It turns out, however, that low-level features at character level, such as character n‑grams, where n ranges from 2 to 4, are among the most effective ones, whereas tapping syntactic or semantic information is less so and may serve only as a complement. Character n‑grams indeed carry various forms of stylistic information, including function word usage, inflections, phonetic preferences, and even word and sentence length distribution dependent on how often white spaces and punctuation occur. Regarding the classification technology applied, the outlined multi-class problems make use of a straightforward classifier, whereas the one-class classification problem of verification requires tailored approaches. One of the best-performing ones includes the reconstruction approach “Unmasking”, which trains a classifier to separate the text passages from the text of unknown authorship from those of the known author, repeating the training iteratively and removing the most discriminative features in each iteration. The decrease of classification performance over iterations is consistently higher if the unknown text has in fact been written by a known author [22]. Besides these notable examples, there are plenty more, many of which have been surveyed in [32]; both for authorship attribution and verification, author profiling as well as reuse detection, dozens of approaches have been proposed over the past two decades. Yet, for all of these tasks, little effort has been spent to develop standardised benchmarks, so that results can hardly be compared across papers. To fill this gap, the PAN workshop for digital text forensics has been initiated, where shared tasks for all of the aforementioned problems have been organised starting 2009. While a complete survey of the results of the PAN initiative is out of the scope of this paper, we refer to the latest overviews of the respective tasks, namely for authorship attribution [19], authorship verification [33], author profiling [28], and the two subtasks of reuse detection, text alignment and source retrieval [15, 27]. These benchmarks have had a significant impact on the community. In a recent large-scale reproducibility study on authorship attribution, they were employed to reimplement and reproduce the 15 most influential approaches from the past two decades, evaluating them on the standardised datasets [26]. The study finds that some of the approaches proposed early on are still competitive with the most recent contributions.

With respect to cyberstalking detection, there are still open challenges in authorship identification that need to be addressed, such as the fact that these technologies do not work well on very short texts, unless many short text from the same author can be gathered. If a stalker sends only very short and only a few well-placed messages, a reliable identification may be circumvented altogether. Moreover, application-dependent style features need to be developed that also take into account the context of the recipient.

## Conclusion and future work

Textual analysis and machine learning are cornerstones towards a technical response to the problem of cyberstalking. This is evident by the different prevention and mitigation techniques discussed in this paper as well as the Anti Cyberstalking Text-based System (ACTS) framework. ACTS’ modules showcase various features to mitigate this type of anti-social offence. By design, it has a prevention mechanism combining the ability to detect, analyse, identify, and block communication. Further, it also has an integrated functionality to quarantine evidence to aid digital forensics investigations. The forensic element of this framework is not limited to logging content but adding a layer of analysis-based metadata to establish relationships between collected evidence, hence supporting investigation. In practice, this capability is also critical to alarm and convince law-enforcement to the severity of the attack as it consequently provides means to assess potential risk.

Our future work in this regard includes the development of a new mechanism to empower users further with evidence-based advice on how to respond to harassment. Victims usually have few choices 1) sending a reply to an unwanted message; 2) ignore it; or 3) outsource the response to a third-party. Some of these actions include further decisions such as deciding the content of the response in the case of sending a reply or identifying a suitable third-party to contact. We argue that machine learning can eventually provide intelligence to guide users towards personalised suitable actions. Accordingly, this ongoing work should also survey existing experiences of victims to support such a system.

Besides personalisation and content analysis, one of the crucial elements of the ACTS framework relies on effective authorship identification in a cyberstalking context. We therefore discussed existing promising approaches for several facets of this challenging task. It becomes clear that the outcome of this line of research can potentially help to detect cyberstalking more accurately. However, most of the approaches have not directly been applied to the problem of cyberstalking detection. Future efforts should therefore focus on applying these mechanisms, potentially in the context of ACTS, directly to the cyberstalking detection problem. One step in this direction could for instance be the organization of a shared task on cyberstalking detection in the context of PAN.

## Footnotes

1
http://www.beds.ac.uk/nccr/.

2 This (controversially discussed) approach means data should be stored only “under court order based on a probable cause” (see also http://www.dw.com/en/germany-calls-for-a-quick-freeze-data-compromise/a-15829029).

3 PAN is an excellence network and workshop series on digital text forensics, where researchers and practitioners study technologies that analyze texts with regard to originality, authorship, and trustworthiness. Almost all of the technologies for corresponding tasks are still in their infancy, and active research is required to push them forward. PAN therefore focuses on the evaluation of selected tasks from the digital text forensics in order to develop large-scale, standardized benchmarks, and to assess the state of the art. PAN has organized shared task events since 2009. See also http://pan.webis.de.

## References

[1] Abbasi A, Chen H (2007) Affect intensity analysis of dark web forums. In: Intelligence and Security Informatics 2007 IEEE. IEEE pp 282–288

[2] Aggarwal S, Burmester M, Henry P, Kermes L, Mulholland J (2005) Anti-cyberstalking: The predator and prey alert (PAPA) system. In: Systematic Approaches to Digital Forensic Engineering, 2005 First International Workshop on. IEEE pp 195–205

[3] Amuchi F, Al-Nemrat A, Alazab M, Layton R (2012) Identifying cyber predators through forensic authorship analysis of chat logs. In: Cybercrime and Trustworthy Computing Workshop (CTC) 2012 Third. IEEE pp 28–37

[4] Burmester M, Henry P, Kermes LS (2005). Tracking cyberstalkers: a cryptographic approach. ACM SIGCAS Comput Soc.

[5] Cappellato L, Ferro N, Jones G, San Juan E (eds) (2015) CLEF 2015 Evaluation Labs and Workshop – Working Notes Papers. In: CEUR Workshop Proceedings 8‑11 September. CEUR-WS.org, Toulouse, France (http://www.clef-initiative.eu/publication/working-notes)

[6] Chang M, Poon CK (2009). Using phrases as features in email classification. J Syst Softw.

[7] Dadvar M, Trieschnigg D, Jong F de (2014) Experts and machines against bullies: A hybrid approach to detect cyberbullies. In: Advances in Artificial Intelligence. Springer pp 275–281

[8] DeSmet A, Bastiaensens S, Van Cleemput K, Poels K, Vandebosch H, De Bourdeaudhuij I (2014). Applying the intervention mapping protocol to the design of a serious game against cyberbullying among young adolescents. Eur Health Psychol.

[9] Dinakar K, Reichart R, Lieberman H (2011). Modeling the detection of textual cyberbullying. The Social Mobile Web.

[10] Fahrnberger G, Nayak D, Martha VS, Ramaswamy S (2014) Safechat: A tool to shield children’s communication from explicit messages. In: Innovations for Community Services (I4CS) 2014 14th International Conference on. IEEE pp 80–86

[11] Ghasem Z, Frommholz I, Maple C, Klas CP, Frommholz I (2015). A machine learning framework to detect and document text-based cyberstalking. Proceedings Information Retrieval Workshop at Lernen-Wissen-Adaptivität (LWA 2015).

[12] Ghasem Z, Frommholz I, Maple C (2015). Machine learning solutions for controlling cyberbullying and cyberstalking. J Inf Secur Res.

[13] Gómez Hidalgo JM, Bringas GC, Sánz EP, García FC (2006) Content based SMS spam filtering. In: Proceedings of the 2006 ACM Symposium on Document Engineering – DocEng ’06. ACM pp 1–8

[14] Gupta A, Kumaraguru P, Sureka A (2012) Characterizing pedophile conversations on the internet using online grooming. Arxiv Prepr (arXiv:1208.4324)

[15] Hagen M, Potthast M, Stein B (2015) Source Retrieval for Plagiarism Detection from Large Web Corpora: Recent Approaches. In: Working Notes Papers of the CLEF 2015 Evaluation Labs, CEUR Workshop Proceedings. CLEF and CEUR-WS.org (http://www.clef-initiative.eu/publication/working-notes)

[16] Haughey H, Epiphaniou G, Al-Khateeb HM (2016). Anonymity networks and the fragile cyber ecosystem. Netw Secur.

[17] Holmes DI (1998). The evolution of stylometry in humanities scholarship. Lit Linguist Comput.

[18] Hsu DF, Marinucci D (2013) Advances in Cyber Security: Technology, Operation, and Experiences. Fordham Univ Press

[19] Juola P (2012) An Overview of the Traditional Authorship Attribution Subtask. In: Forner P, Karlgren J, Womser-Hacker C (eds) CLEF 2012 Evaluation Labs and Workshop – Working Notes Papers 17–20 September. Rome, Italy (http://www.clef-initiative.eu/publication/working-notes)

[20] al Khateeb HM, Epiphaniou G (2016). How technology can mitigate and counteract cyber-stalking and online grooming. Comput Fraud Secur.

[21] Koppel M, Schler J (2004). Authorship verification as a one-class classification problem. Proceedings of the Twenty-first International Conference on Machine Learning, ICML ’04.

[22] Koppel M, Schler J, Bonchek-Dokow E (2007). Measuring differentiability: Unmasking pseudonymous authors. J Mach Learn Res.

[23] Maple C, Short E, Brown A, Bryden C, Salter M (2012). Cyberstalking in the UK: Analysis and recommendations. Int J Distributed Syst Technol (ijdst).

[24] Mendenhall TC (1887). The characteristic curves of composition. Science.

[25] NCCR (2015) A Practical Guide To Coping With Cyberstalking. National Centre for Cyberstalking Research, Andrews UK Limited

[26] Potthast M, Braun S, Buz T, Duffhauss F, Friedrich F, Gülzow J, Köhler J, Lötzsch W, Müller F, Müller M, Paßmann R, Reinke B, Rettenmeier L, Rometsch T, Sommer T, Träger M, Wilhelm S, Stein B, Stamatatos E, Hagen M, Ferro N, Crestani F, Moens MF, Mothe J, Silvestri F, Di Nunzio G, Hauff C, Silvello G (2016). Who Wrote the Web? Revisiting Influential Author Identification Research Applicable to Information Retrieval. Advances in Information Retrieval. 38th European Conference on IR Resarch (ECIR 16).

[27] Potthast M, Göring S, Rosso P, Stein B (2015) Towards Data Submissions for Shared Tasks: First Experiences for the Task of Text Alignment. In: Working Notes Papers of the CLEF 2015 Evaluation Labs, CEUR Workshop Proceedings. CLEF and CEUR-WS.org (http://www.clef-initiative.eu/publication/working-notes)

[28] Rangel F, Celli F, Rosso P, Potthast M, Stein B, Daelemans W (2015). Overview of the 3rd Author Profiling Task at PAN 2015. Cappellato et al. [5.

[29] Satta R, Stirparo P (2013). Picture-to-identity linking of social network accounts based on sensor pattern noise.

[30] Sebastiani F (2002). Machine learning in automated text categorization. ACM Comput Surv.

[31] Short E, Linford S, Wheatcroft JM, Maple C (2014). The impact of cyberstalking: The lived experience-a thematic analysis. Stud Health Technol Inform.

[32] Stamatatos E (2009). A survey of modern authorship attribution methods. J Am Soc Inf Sci Technol.

[33] Stamatatos E, Daelemans W, Verhoeven B, Juola P, López-López A, Potthast M, Stein B (2015). Overview of the Author Identification Task at PAN 2015. Cappellato et al. [5].

[34] Stein B, Lipka N, Prettenhofer P (2011). Intrinsic Plagiarism Analysis. Lang Resour Eval.

[35] Tax DMJ (2001) One-class classification. Ph.D. thesis

[36] The Crown Prosecution Service Guidelines on prosecuting cases involving communications sent via social media. http://www.cps.gov.uk/legal/a_to_c/communications_sent_via_social_media/. Accessed 2016-04-12

[37] UK Cabinet Office The UK Cyber Security Strategy – protecting and promoting the UK in a digital world. https://www.gov.uk/government/uploads/system/uploads/attachment_data/file/60961/uk-cyber-security-strategy-final.pdf. Accessed 2016-01-29

[38] Zheng R, Li J, Chen H, Huang Z (2006). A framework for authorship identification of online messages: Writing-style features and classification techniques. J Am Soc Gor Inf Sci Technol.

[39] Van der Zwaan J, Dignum M, Jonker C (2010) Simulating peer support for victims of cyberbullying. In: BNAIC 2010: 22rd Benelux Conference on Artificial Intelligence 25–26 October 2010. Luxembourg

